# Overexpression of ORMDL3 confers sexual dimorphism in diet-induced non-alcoholic steatohepatitis

**DOI:** 10.1016/j.molmet.2023.101851

**Published:** 2023-12-09

**Authors:** Ryan D.R. Brown, Christopher D. Green, Cynthia Weigel, Bin Ni, Francesco S. Celi, Richard L. Proia, Sarah Spiegel

**Affiliations:** 1 Department of Biochemistry and Molecular Biology, Virginia Commonwealth University School of Medicine, Richmond, VA, USA; 2 Department of Internal Medicine, Virginia Commonwealth University School of Medicine, Richmond, VA, USA; 3 Genetics and Biochemistry Branch, National Institute of Diabetes and Digestive and Kidney Diseases, NIH, Bethesda, MD, USA

**Keywords:** NASH, Hepatic steatosis, Fibrosis, Sphingolipids, ORMDL, Adipose

## Abstract

**Objective:**

The bioactive sphingolipid metabolites ceramide and sphingosine-1-phosphate (S1P) accumulate with overnutrition and have been implicated in non-alcoholic steatohepatitis (NASH) development. ORMDL3, a negative regulator of the rate-limiting step in ceramide biosynthesis, has been identified as an obesity-related gene. Therefore, we assessed the role of ORMDL3 in diet-induced obesity and development of NASH.

**Methods:**

Globally overexpressing *Ormdl3-Flag* transgenic mice (ORMDL3^TG^) were fed a western high-fat, carbohydrate and cholesterol enriched diet, with high fructose-glucose drinking water. Physiological, biochemical and sphingolipidomic analyses were employed to measure the effect of ORMDL3 overexpression on NASH development.

**Results:**

ORMDL3^TG^ male but not female mice fed a western high-fat diet and sugar water had exacerbated adipocyte hypertrophy together with increased severity of white adipose inflammation and fibrosis. Hepatic steatosis, dyslipidemia, impaired glucose homeostasis, hyperinsulinemia, and insulin resistance were significantly more severe only in obese ORMDL3^TG^ male mice that accompanied dramatic liver fibrosis, inflammation, and formation of hepatic crown-like structures, which are unique features of human and murine NASH. Obesogenic diet induces ORMDL expression in male mice but reduces it in females. Mechanistically, overexpression of *Ormdl3* lowered the levels of S1P and ceramides only in obese female mice and antithetically increased them in tissues of obese males. ORMDL3^TG^ male mice exhibited a much greater induction of the UPR, propagating ER stress that contributed to their early development of NASH.

**Conclusions:**

This study uncovered a previously unrecognized role for ORMDL3 in sexual dimorphism important for the development and progression of NASH.

## Highlights

• High ORMDL3 overexpression in male but not female mice fed an obesogenic diet led to earlier NASH development.
• ORMDL3 overexpression increased ceramide and S1P as well as hepatic ER stress in obese male mice.
• Obesogenic diet induces ORMDL expression in male mice but reduces it in females.
• A novel role of ORMDL3 in sexual dimorphism that is important for NASH development.
• Increased ORMDL3 could be relevant to the greater incidence of NASH in men than in women.

## Graphical abstract

**Figure:**
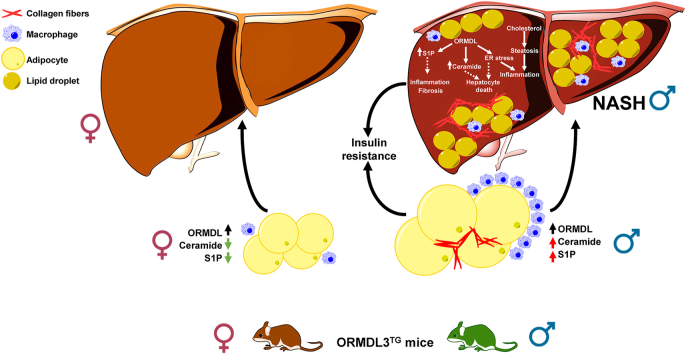

## 1 Introduction

Obesity is now considered to be a global crisis and approximately 30% consuming a ‘western diet’ high in fat and sugar will develop non-alcoholic fatty liver disease (NAFLD) [1,2]. NAFLD is the leading cause of chronic liver disease worldwide, ranging from steatosis (ectopic lipid accumulation) to non-alcoholic steatohepatitis (NASH), with progressive fibrosis [[1], [2], [3]]. Although frequently clinically silent, NASH can gradually progress to cirrhosis, end-stage liver disease, and hepatocellular carcinoma [1,4]. Overnutrition-induced lipotoxicity, hepatic steatosis, and chronic low-grade inflammation lead to liver oxidative stress and endoplasmic reticulum (ER) stress, which further progress to NASH with the appearance of hepatocellular injury and fibrosis [4]. NASH is a sex-dimorphic disease, with a greater incidence in men than in women. Yet these sex differences are still not well understood [5].

The bioactive sphingolipid metabolites ceramide and sphingosine-1-phosphate (S1P) are another addition to lipids such as triglycerides and cholesterol that accumulate with overnutrition, and their dysregulation has been shown to correlate with obesity, steatosis, and the development of murine and human NASH [[6], [7], [8], [9], [10], [11]]. Indeed, inhibition of serine palmitoyltransferase (SPT), the rate-limiting step in ceramide biosynthesis, or deletion of its subunits *Sptlc1* or *Sptlc2* in obese mice limits the formation of these obesogenic sphingolipids and consequently reduces diet-induced obesity, improves glucose tolerance and insulin resistance, preventing the progression to NASH [[12], [13], [14]]. The three ORMDL isoforms (ORMDL1-3) are components of the SPT complex that negatively regulate SPT activity [15]. Recently, attention has been focused on ORMDL sphingolipid biosynthesis regulator 3 (*ORMDL3*), which has been identified as an obesity-related gene that negatively correlates with the body mass index [[16], [17], [18]]. However, little is known about its involvement in obesity-associated pathologies. Although *Ormdl3* was reported to be upregulated in the β-cells of *ob/ob* mice that spontaneously develop obesity [16], another study showed that loss of Ormdl3 in β-cells did not alter glucose tolerance or insulin sensitivity [19]. Conversely, global deletion of *Ormdl3* in mice fed an obesogenic diet increases weight gain and insulin resistance [18]. These contradictory studies suggest that a better understanding of the role of ORMDL3 in metabolic functions is required.

To examine the potential role of ORMDL3 in metabolically active tissues and the development of NASH, we utilized *Ormdl3* overexpressing transgenic mice (ORMDL3^TG^) fed a Western high-fat diet and sugar water (HFD/SW), previously shown to mimic a ‘western diet’ and promote the development of obesity, hepatic steatosis and dyslipidemia [20]. Our study uncovered a novel role of ORMDL3 in sexual dimorphism that is important for the development and progression of NASH, and highlights the sexually dimorphic nature of NASH and its link with fibrosis in humans.

## 2 Material and methods

### 2.1 Animal

*Ormdl3* transgenic C57BL/6J mice were generated by knocking in an *Ormdl3* transgene via homologous recombination to the Rosa26 locus in embryonic stem cells, as previously described [21]. Heterozygous *Ormdl3* transgenic mice were inbred to generate transgenic and control littermates. Mice were weaned at 3–4 weeks of age and maintained on either standard rodent chow (Harlan TD.7912, Envigo) in addition to water, or high-fat diet (HFD) (42% kcal from milk fat, 42.7% kcal from sucrose-enriched carbohydrates, 15.2% kcal from protein and 0.2% cholesterol) (Harlan TD.88137, Envigo) in addition to sugar water (SW) (23.1 g/L D-fructose and 18.9 g/L D-glucose) *ad libitum*. Mice were housed in the animal care facility at Virginia Commonwealth University under regulated temperature (21°C–23°C), humidity (50%–60%) and 12-hour dark/light cycles. All animal protocols and procedures were approved by the Virginia Commonwealth University Institutional Animal Care and Use committee.

### 2.2 Body composition analysis

Mice were weighed, restrained and body composition measured using the LF90II time-domain NMR minispec (Bruker Optik, Massachusetts) to obtain fat and lean mass.

### 2.3 Whole-body metabolic phenotyping

Mice were acclimated 24 h in individual metabolic chambers of the PhenoMaster (TSE Systems, Germany) under regulated temperature (23 °C), humidity (50%) and 12-hour dark/light cycles (70% light intensity). VO_2_, VCO_2_, energy expenditure (EE), respiratory exchange ratio (RER), locomotor activity (beam breaks; X + Y planes), food consumption and water intake were then measured for 6 consecutive days. Raw data was exported from the PhenoMaster and graphed using the publicly available indirect calorimetry analysis tool (CalR; https://CalRapp.org/).

### 2.4 Tissue and plasma analysis

Mice were fasted for 6 h (7AM–1PM), euthanized by 5% isoflurane inhalation, blood drawn via cardiac puncture with heparinized syringes and perfused transcardially with PBS supplemented with 0.1% heparin (#375095-500KU, Sigma). Plasma was collected by centrifugation of blood at 2,000×*g* for 15 min at 4 °C. Liver, brown, gonadal and inguinal subcutaneous adipose tissues pads were weighed and snap-frozen in liquid N_2_ or processed for embedding. Circulating plasma concentrations of triglycerides, cholesterol, non-esterified fatty acids (NEFA) and phospholipids were measured by the University of Cincinnati Mouse Metabolic Phenotyping Center and insulin by ELISA (#90080, CrystalChem). Circulating insulin and blood glucose from mice fasted for 6 h was subsequently used to calculate the homeostatic model assessment for insulin resistance (HOMA-IR) by the equation: (fasted insulin (ng/ml)x fasted glucose (mM))/22.5. For quantification of liver triglycerides, 40 mg of snap-frozen pulverised liver tissue was saponified in six volumes of 2:1 ethanol:30% KOH at 60 °C for 5 h 1.08 volumes of 1 M MgCl_2_ were added to the tissue and incubated on ice for 10 min before being centrifuged and the supernatant analyzed as recommended by the manufacturer (#SB2200225, Fisher Scientific). For quantification of liver cholesterol (#J3190, Promega), 10 mg of snap-frozen liver tissue was briefly homogenised in 400 μL PBS on ice, diluted 1:5 in PBS, and then diluted 1:50 in cholesterol lysis solution. Aliquots were taken for BCA analysis and 20 μL of standard or sample incubated for 30 min at 37 °C. 20 μL detection reagent was added to each well and incubated for 1 h at room temperature. Luminescence was measured with the VictorX4 luminescence detector (#2030-0040, Perkin Elmer) and normalized to protein content.

### 2.5 Intraperitoneal glucose tolerance testing

Mice were fasted for 6 h (7AM–1PM) for glucose tolerance test (GTT). Baseline blood glucose was measured from a nick in the tail vein using a Nipro TRUEtrack Blood Glucose Meter (#B0727V3XQX, Amazon). Glucose (2 mg/g body weight in sterile PBS) was then injected intraperitoneally, and blood glucose measured at the indicated times post-injection.

### 2.6 Immunofluorescence

For immunofluorescence staining with F4/80, paraffin sections of adipose were dewaxed and rehydrated by submerging twice in Histoclear, 90% Histoclear/10% ethanol, twice in 100% ethanol, twice in 95% ethanol, twice in 80% ethanol, twice in 70% ethanol all for 5 min and finally twice in distilled water for 20 min. Slides were then submerged in 98 °C HIER antigen-retrieval buffer (#ab208572, Abcam) for 20 min. Sections were washed 3 times in PBS for 5 min and fixed in ice-cold methanol for 15 min at −20 °C before further washing in PBS 3 times for 10 min each. Sections were blocked and permeabilised with PBS containing 5% normal goat serum (#31872, ThermoFisher), 1% BSA and 0.4% Triton X-100 (buffer A) for 1 h at room temperature. Slides were subsequently incubated overnight with anti-F4/80 (#157307, BioLegend) diluted 1:200 in buffer A at 4 °C. Slides were washed 3 times with PBS and probed with anti-mouse AlexaFluor 555 (#A28180, ThermoFisher) diluted 1:200 in buffer A at room temperature for 1 h. Slides were then washed 3 times with PBS for 5 min and mounted with VECTASHIELD Vibrance antifade mounting medium containing DAPI (#H-1800, Vector Laboratories). Images were captured using a BZ-X810 fluorescence microscope (#BZ-X810, Keyence). Color deconvolution by ImageJ was used to measure the positive areas of F4/80-stained images. The number of CLS were counted in the whole area of each F4/80-stained section and expressed as the mean numbers per mm^2^.

### 2.7 Western blotting

Equal weights of tissue were homogenised in RIPA buffer (50 mM Tris-HCl; pH 7.4, 1 mM EDTA, 150 mM NaCl, 0.1% Sodium dodecyl sulphate, 1% Triton X-100, 0.5% sodium deoxycholate) containing HALT protease/phosphatase inhibitors (#78440, ThermoFisher) using a Dounce homogeniser for approximately 40 strokes or until visibly homogenised and left on ice for 30 min. Liver homogenates were then centrifuged at 12,000 xG for 15 min at 4 °C and the supernatant collected. Adipose homogenates were centrifuged at 3,000 xG for 5 min at 4 °C, the bottom phase was further centrifuged at 12,000 xG for 15 min at 4 °C, and the supernatant collected. Proteins were measured with the Piece BCA Protein Assay Kit (#23227, ThermoFisher). Equal amounts of protein were separated by 10% SDS-PAGE and transferred to 0.2 μm pore-size nitrocellulose (#1620112, BioRad) using the PierceG2 Fast Blotter (#62287, ThermoFisher). Membranes were then blocked, and blots were incubated with the following primary antibodies: anti-ORMDL3 (1:1000; #ABN417, Sigma, RRID:AB_2943375), anti-FLAG (1:1000; #F1804, Sigma, RRID:AB_262044), anti-uncoupling protein-1 (UCP1) (1:1000; #14670, CST, RRID:AB_2687530), anti-ATF6 (1:1000, #NBP1-40256, Novus Biologicals, RRID:AB_2058774), anti-IRE1 (1:1000, #3294, CST, RRID:AB_823545), anti-phospho-IRE1 (Ser724) (1:1000, #PA5-105424, Invitrogen, RRID:AB_2816852), anti-eIF2α (1:1000, #9722, CST, RRID:AB_2230924), anti-phospho-eIF2a (Ser51) (1:1000, #9721, CST, RRID:AB_330951), anti-CHOP (1:1000, #2895, CST, RRID:AB_2089524), anti-vinculin (1:1000, #4650, CST, RRID:AB_10559207), anti-xbp-1s.

(1:1000, #40435, CST, RRID:AB_2891025), anti-b-tubulin (1:2000; #2146, CST, RRID:AB_2210545). and anti-b-actin (1:1000; #3700, CST, RRID:AB_2242334). Peroxidase-conjugated goat anti-rabbit secondary antibody (1:5000; #NC9734651, FisherScientific) or Peroxidase-conjugated goat anti-mouse secondary antibody (1:5000; #NC9994806, FisherScientific) were used and protein bands visualized using chemiluminescent substrate (#34578, ThermoFisher). Densitometric quantification of immunoblot bands normalized to loading controls was by ImageJ.

### 2.8 Histology

Adipose or liver tissues were fixed in 10% neutral buffered formalin (#245684, FisherScientific) for 24 h and embedded in paraffin or subsequently infiltrated with 30% sucrose for a further 48 h before being frozen in Tissue-Tek optimal cutting temperature solution (#4583, Sakura). Paraffin sections were stained for H&E, or αSMA by Virgnia Commonwealth University Tissue and Data Acquisition and Analysis Core. For staining of collagen fibres, paraffin sections were dewaxed and rehydrated by submerging in Histoclear (#5089990147, FisherScientific), Histoclear 1:1 with ethanol then ethanol for 10 min, 50% ethanol/distilled water then distilled water each for 5 min. Sections were then stained with Sirius red/Fast green (#9046, Chondrex). Frozen liver sections were also stained with oil red O (#O0625, Sigma) to assess neutral lipid accumulation as previously described [22]. Color deconvolution by ImageJ was used to measure the positive areas of Sirius red, αSMA, and oil red O stained images.

### 2.9 Adipocyte size determination

Brightfield images of H&E-stained sections were captured with a BZ-X810 fluorescence microscope (#BZ-X810, Keyence) and adipocyte size was automatically calculated from at least 760 adipocytes per sample using the Adiposoft plugin [23] for ImageJ.

### 2.10 Quantification of sphingolipids by mass spectrometry

Lipids were extracted from tissues and sphingolipids quantified by liquid chromatography electrospray ionization-tandem mass spectrometry (LC-ESI-MS/MS; API 5500 QTRAP; ABSciex) as previously described [21].

### 2.11 Mouse genotyping

Genomic DNA was isolated from tails as previously described [24] and 3 μL used for PCR with GoTaq Master Mix (#M712C, Promega) with the following primers: ROSA26; GCACGTTTCCGACTTGAGTTGCC and CAACGCCCACACACCAGGTTAG; BGH polyA; TGCCACTCCCACTGTCCTTTCC. 15 μL of PCR product was separated on a 2% agarose gel. The band sizes of 300 and 392 base pairs indicate presence of the transgene and the wild-type ROSA26 locus, respectively.

### 2.12 RNA isolation and qPCR

RNA was extracted from liver and adipose tissues using TRIzol (#15596026, ThermoFisher) and concentration measured with the NanoDrop 2000 (#ND2000, ThermoFisher). 2 μg was treated with RQ1 DNase (#M6101, Promega) and converted to cDNA with High-Capacity cDNA Reverse Transcription Kit (#4368814, ThermoFisher). mRNA expression was determined by mixing cDNA with the following primer pairs (5′-3′): *Acta2* for GTGTGAAGAGGAAGACAGCAC and rev GTGATGATGCCGTGTTCTATCG, *col1a1* for TCAGCCACCTCAAGAGAAGTC and rev CTCCGGATGTTCTCAATCTGC, or with pre made primers for *Mcp1* (#qMmuCED0048300, BioRad). Products were measured with a CFX Opus 96 Real-Time PCR Detection System (#12011319, BioRad) and relative changes in gene expression were determined by ΔΔC_t_ normalized to TATA-binding protein.

### 2.13 Statistical analysis

Statistical significance using GraphPad Prism 8 software was calculated with two-tailed Student's t-test for comparison of two groups, or with ANOVA followed by post hoc tests for multiple comparisons. Two-way ANOVA (factors: diet and genotype, or diet and gender) followed by Tukey's post-hoc test for multiple comparisons to evaluate significant effects of factors and interactions when appropriate (Supplemental Table 1). For all experiments, the normality of the data from each group was first checked using the Shapiro-Wilk test. The designations for significance levels are ∗p < 0.05, ∗∗p < 0.01, ∗∗∗p < 0.001 and ∗∗∗∗p < 0.0001 The same significance values also apply to ^#^.

## 3 Results

### 3.1 Lack of effect of global overexpression of *Ormdl3* in mice on adipose tissue thermogenesis

**Figure 1:**
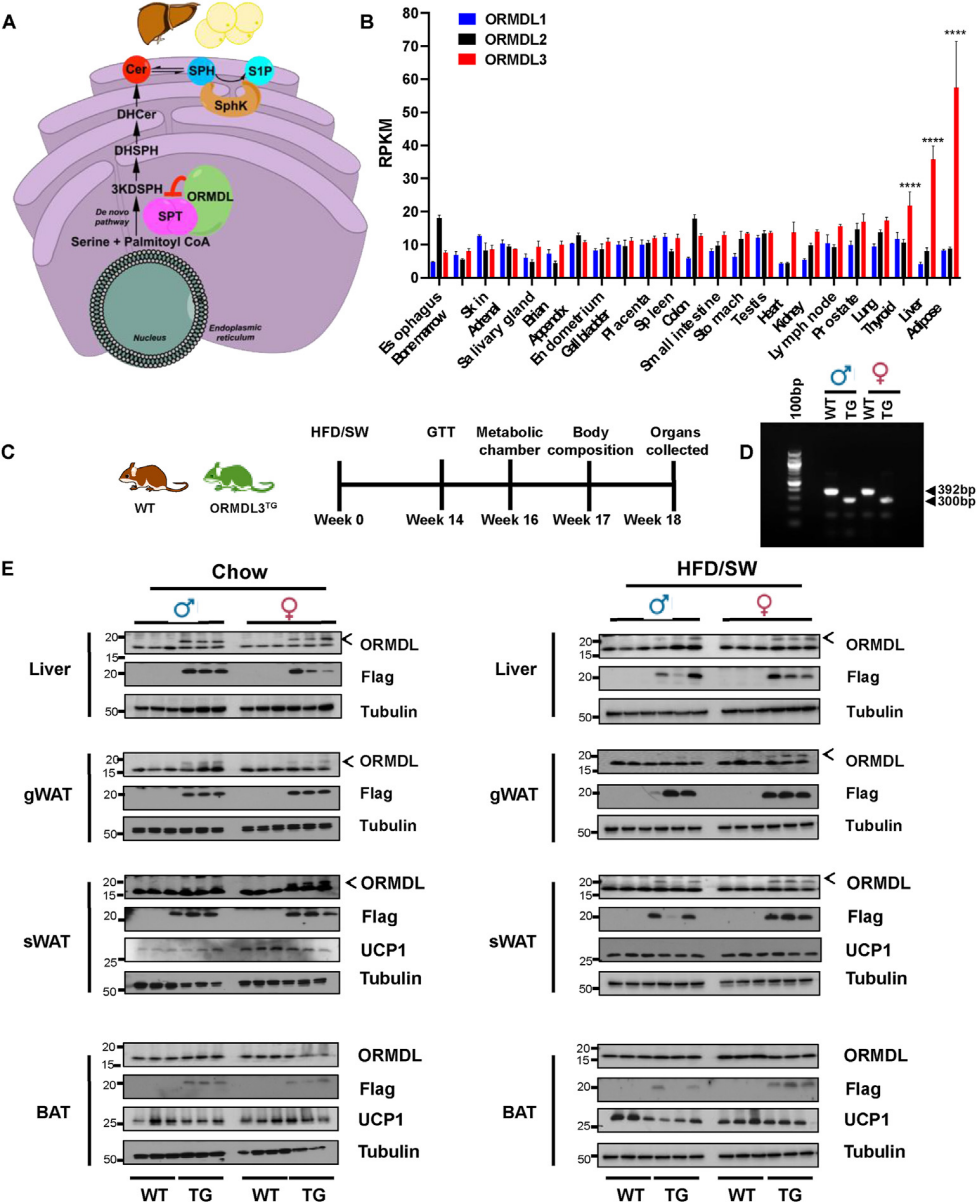
**Lack of effect of global overexpression of *Ormdl3* in mice on adipose tissue thermogenesis.** (A) Scheme illustrating negative regulation of serine palmitoyltransferase by Ormdl3 to limit sphingolipid biosynthesis. (B) RNA-seq of ORMDL3 in healthy human patient tissue (PRJEB4337) (N = 3–7). Data are mean ± SEM, ∗∗∗∗p ≤ 0.0001 compared to esophagus. two-way analysis of variance test followed by Dunnett's multiple comparisons test. (C) Scheme depicting the model of 18-week standard chow or high-fat diet with sugar water feeding (HFD/SW) and the time points to measure glucose tolerance test (GTT), metabolic phenotyping and body composition. (D) DNA agarose gel showing the insertion of the *ORMDL3*-FLAG transgene (TG) compared to WT mice (WT). (E) Western blots of liver, gonadal adipose (gWAT), subcutaneous adipose (sWAT), or brown adipose tissue (BAT) from chow and HFD/SW fed mice. Proteins were visualized with anti-FLAG, anti-ORMDL or anti-UCP1 antibodies. Tubulin was used as loading controls (N = 3). Arrowheads indicate ORMDL3-FLAG. Densitometric analyses of blots in (E) are shown in Supplemental Fig. 2.

*ORMDL3* gene polymorphism has been associated with type 1 diabetes [25] and was suggested to be an obesity-related gene, whereby its expression negatively correlated with body mass index BMI [[16], [17], [18]]. Moreover, analysis of publicly available data (BioProject: PRJEB4337 from [26]) revealed that tissue expression of *Ormdl3* but not *Ormdl1* or *Ormdl2* in humans is highest in metabolically active organs such as the liver and adipose tissue (Figure 1B). Likewise, and consistent with a previous study [27], ORMDLs expression in mice was abundant in liver and adipose tissues, and relatively low in lung and spleen (Supplemental Fig. 1). Therefore, we sought to assess the role of ORMDL3 in obesity. To this end, globally overexpressing *Ormdl3-Flag* transgenic mice (ORMDL3^TG^) were fed a high fat, high carbohydrate with cholesterol diet (HFD), and high fructose-glucose drinking water (sugar water, SW) for 18 weeks (Figure 1C), which was previously shown to mimic a typical western diet and induce the development of obesity, fatty liver, and dyslipidemia [20]. The *Ormdl3* transgene was confirmed by PCR using genomic DNA separated by agarose gel electrophoresis (Figure 1D). There are no antibodies that are specific for ORMDL3, as the ORMDL3 protein shares over 80% homology with ORMDL1 and ORMDL2. The FLAG-tag epitope verified the specific detection of the overexpressed ORMDL3 protein using an anti-Flag antibody (Figure 1E). Overexpression of ORMDL3-FLAG was evident in the liver, visceral gonadal white adipose tissue (gWAT), and subcutaneous inguinal white adipose tissue (sWAT) depots (Figure 1E and Supplemental Fig. 2). However, minimal overexpression of ORMDL3 was detected in brown adipose tissue (BAT), which dissipates energy by generating heat through uncoupled respiration mediated by uncoupling protein-1 (UCP1) [28] (Figure 1E). Since it was recently reported that depletion of *Ormdl3* impairs thermogenic function of BAT [18], we examined the effects of *Ormdl3* overexpression on UCP1. UCP1 levels were similar in WT and *Ormdl3* transgenic sWAT, which contains beige adipocytes that conditionally express UCP1. Moreover, *Ormdl3* overexpression did not induce major changes in UCP1 levels in BAT from mice fed chow or HFD/SW diet (Figure 1E). In addition, Ormdl3 overexpression did not elicit statistically significant changes in rectal body temperature in ether male or female mice (Supplemental Fig. 3), supporting lack of thermogenetic effects. This is likely due to the low overexpression of ORMDL3-FLAG in BAT (Figure 1E and Supplemental Fig. 2); hence, the major focus was subsequently on white adipose tissue and the liver.

### 3.2 Ormdl3 overexpression promotes glucose intolerance and insulin resistance in male mice without affecting diet-induced weight gain

**Figure 2:**
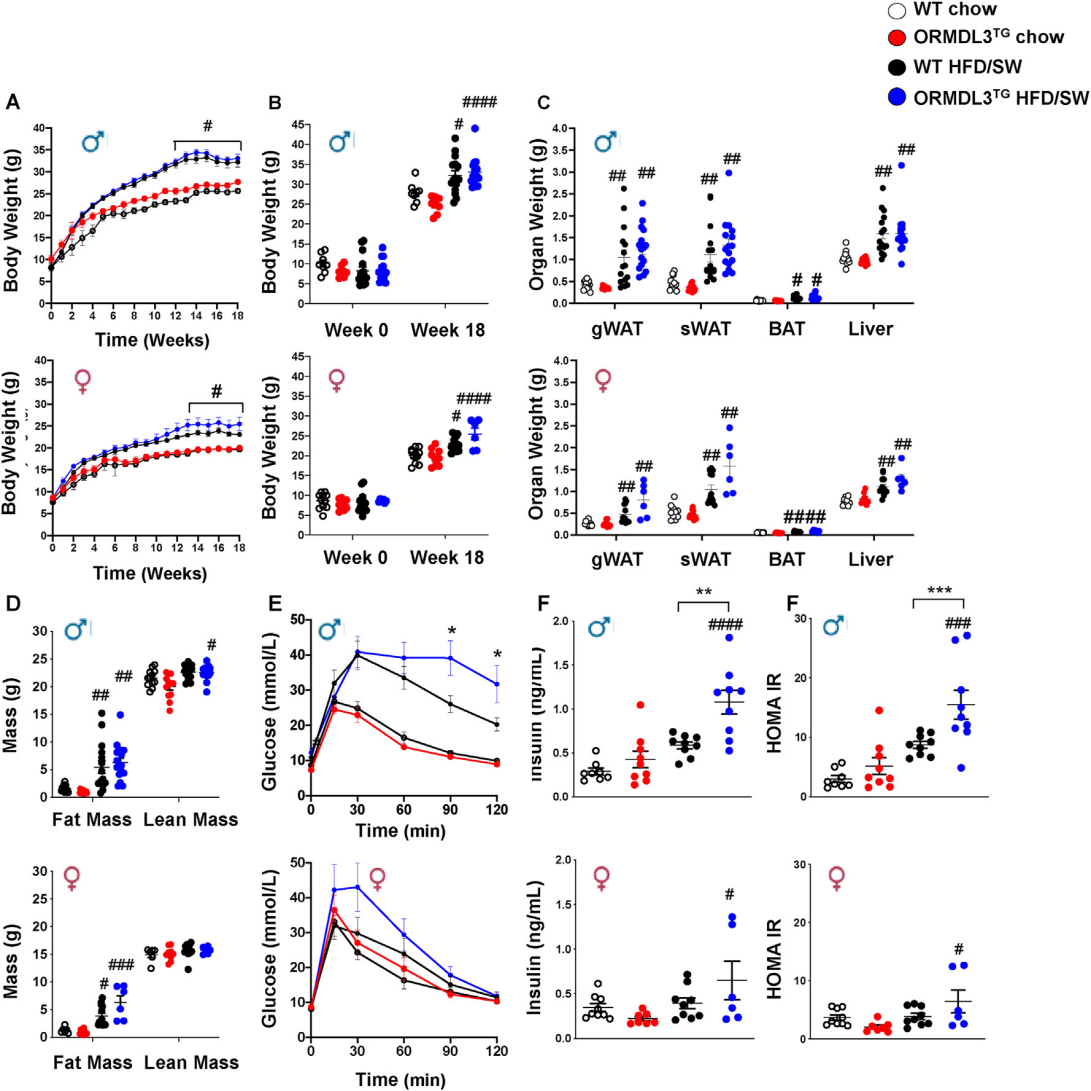
***Ormdl3* overexpression has no effect on diet-induced weight gain but increases glucose intolerance and insulin resistance in male mice.** WT and ORMDL3^TG^ male and female mice were fed chow or high fat and cholesterol diet with high fructose/glucose (HFD/SW) for 18 weeks. (A) Body weight increase with weeks on diet (B) Body weight at the beginning and end of diet (N = 8,8, 15,16 for male, N = 11,9,13,6 for female). (A) Note that due to similarity in weights, some lines overlap and some error bars are smaller than symbols. (A,B) Three-way analysis of variance test followed by Tukey's multiple comparison test, Data are mean ± SEM, ^#^p ≤ 0.05, compared to corresponding littermate controls fed a chow diet. (C) Mass of gonadal white adipose (gWAT), subcutaneous white adipose (sWAT), brown adipose (BAT) and liver. (D) Body composition determined by fat and lean mass. (C, D) (N = 12,12, 15,16 for male, N = 10,10,13,6 for female). (E) Glucose tolerance (GTT) (N = 12,13, 15,15 for male, N = 15,13,13,6 for female). (F) Fasting plasma insulin concentration. (G) HOMA-IR. (F,G) (N = 8,9,9,9 for male, N = 9,7,9,6 for female). (C–G) ^#^p ≤ 0.05, compared to corresponding littermate controls fed a chow diet. ∗p ≤ 0.05, compared to WT fed HFD/SW. Two-way analysis of variance test followed by Tukey's multiple comparison test.

Previous reports have shown that dietary supplementation with myriocin, an SPT inhibitor, inhibits diet-induced obesity in mice fed an obesogenic diet [12]. Therefore, we examined the effects of overexpressing Ormdl3, which negatively regulates SPT activity, in diet-induced weight gain. No significant differences in weight gain or tissue weights were observed between WT and ORMDL3^TG^ mice on chow diet (Figure 2). Both male and female mice fed a Western diet gained significant weight compared to their chow-fed littermates, with greater weight gain in males than in females (Figure 2A,B and Supplemental Table 1) but neither a genotype effect nor diet x genotype interaction were observed, suggesting that, *Ormdl3* overexpression did not significantly influence diet-induced obesity in either gender (Figure 2A,B). *Ormdl3* overexpression also did not significantly alter total fat mass, subcutaneous and visceral adipose depots, or liver weights compared to those of WT mice (Figure 2B,C). There were also no differences in food intake, water consumption, locomotor activity, energy expenditure, oxygen consumption, carbon dioxide (VCO_2_) production or respiratory exchange ratio (RER) between ORMDL3^TG^ and WT mice (Supplemental Fig. 4).

As inhibition or deletion of SPT improved glucose tolerance in obese mice [12], we next investigated whether *Ormdl3* overexpression could improve whole-body glucose metabolism. Surprisingly, although there were no differences in chow fed mice, the Western diet fed ORMDL3^TG^ male, but not female, mice had impaired glucose tolerance and markedly increased circulating insulin levels, as well as an increase in homeostatic model assessment of insulin resistance (HOMA-IR) (Figure 2D–F), indicating low insulin sensitivity compared to WT littermates fed a Western diet. These data suggest that *Ormdl3* overexpression only in males promotes glucose intolerance without causing significant weight gain.

### 3.3 Visceral adipose of obese ORMDL3^TG^ male mice displays severe adipocyte hypertrophy, inflammation, and fibrosis

**Figure 3:**
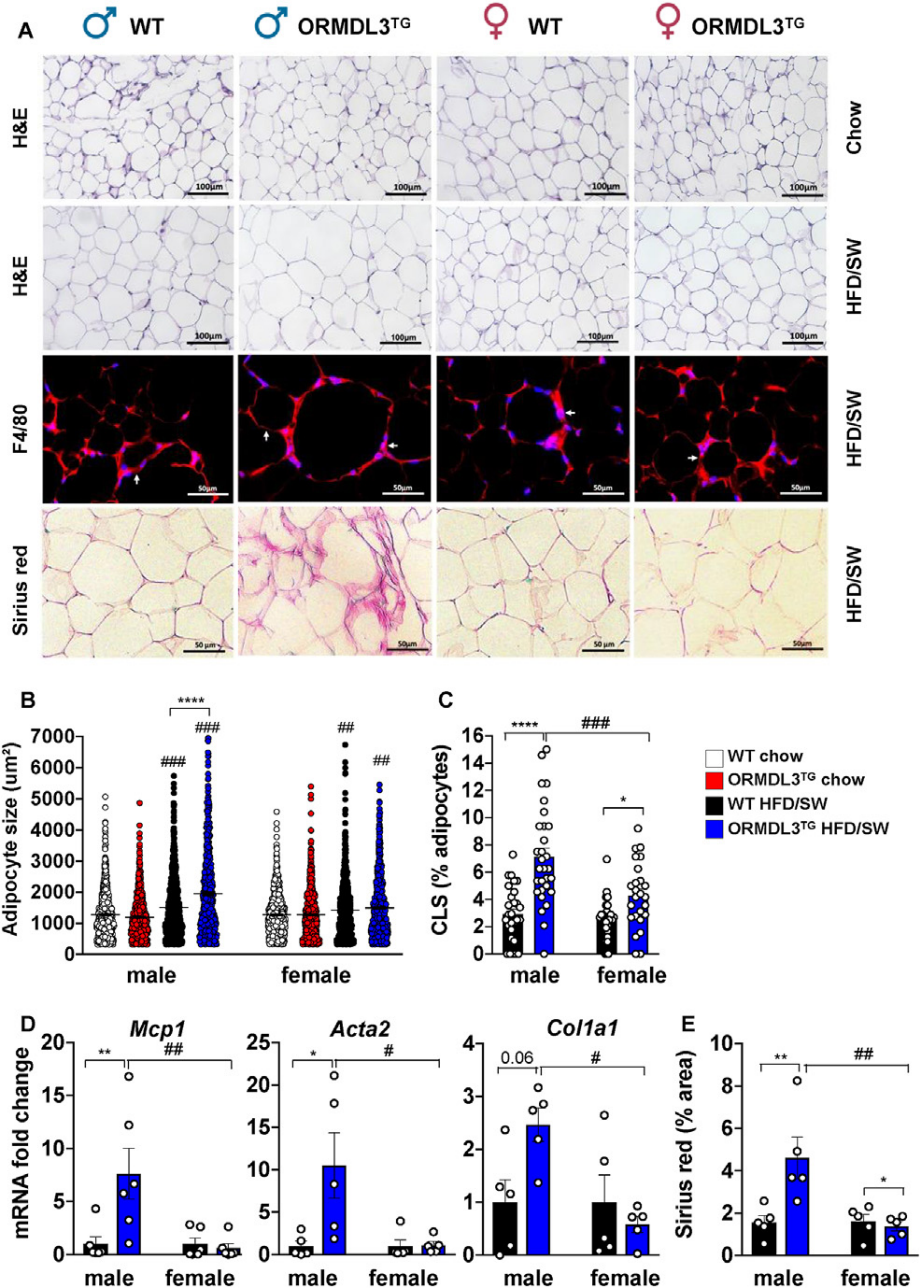
**Increased adipocyte hypertrophy, inflammation, and worsened fibrosis in visceral adipose of obese male ORMDL3**^**TG**^**mice. (A**–**E)** WT and ORMDL3^TG^ male and female mice were fed chow or HFD/SW for 18 weeks as indicated. (N = 5 mice per group). (A) Representative images of hematoxylin-eosin (H&E), Sirius Red, and F4/80-stained gonadal white adipose tissue (gWAT). (B) Adipocyte cell size. Data are mean ± SEM, ^#^p ≤ 0.05 compared to corresponding littermate controls fed a chow diet. ∗p ≤ 0.05 compared to WT fed HFD/SW. One-way analysis of variance test followed by Tukey's multiple comparison test. (C) Percent of crown-like structures (CLS). (N = 5 mice per group with at least 25–30 different fields numerated). (D) mRNA expression of *mcp-1* and fibrotic markers in gWAT. (E) Fibrosis quantified as percent red staining/total tissue surface area. (N = 5 mice per group). (C–E) ∗p ≤ 0.05 compared to WT fed HFD/SW, ^#^p ≤ 0.05 compared to male ORMDL3^TG^. Two-way analysis of variance test followed by Tukey's multiple comparison test.

Because obese male ORMDL3^TG^ mice had increased glucose intolerance (Figure 2D), and insulin resistance correlates with adipocyte hypertrophy, chronic inflammation, and fibrosis [29], we asked whether overexpression of ORMDL3 influenced adipose remodelling. No major differences in gonadal adipocyte size were noted in mice fed chow diet due to ORMDL3 overexpression (Figure 3A,B). Histological analysis also showed that the obesogenic diet increased the size of gonadal adipocytes, and *Ormdl3* overexpression in males markedly further augmented their sizes (Figure 3A,B). However, the HFD/SW diet induced a smaller increase in the size of female adipocytes and *Ormdl3* overexpression did not increase it further (Figure 3A,B).

Inflammation and macrophage infiltration into adipose depots are associated with adipocyte hypertrophy and link obesity with insulin resistance [29]. Thus, we immunostained for the cell-surface macrophage marker F4/80 to detect macrophages infiltrating the visceral adipose tissue of obese mice that encircled dying/dead adipocytes, forming characteristic macrophage-laden crown-like structures (CLS) [30]. There was a significantly increased number of CLS in HFD/SW-fed ORMDL3^TG^ male mice compared to their obese WT littermates, with a much smaller relative increase in CLS in ORMDL3^TG^ female mice (Figure 3C). This is consistent with the increased expression of the chemokine Mcp1 known to recruit macrophages, which was only observed in obese ORMDL3^TG^ males (Figure 3D). Adipose tissue fibrosis detected by Sirius Red staining is often associated with adipocyte death and macrophage infiltration [31]. gWAT from obese ORMDL3^TG^ male mice, but not those from female mice, exhibited substantially greater Sirius Red staining than sex-matched obese WT mice (Figure 3E). This correlated with significantly increased fibrogenic activity in obese ORMDL3^TG^ male mice, as measured by mRNA expression of the pro-fibrotic genes collagen type I alpha 1 chain (*Col1a1*) and alpha smooth muscle actin (*Acta2*) (Figure 3D). Similarly, more severe adipocyte hypertrophy, accompanied by increased collagen deposition, was present in the subcutaneous fat (sWAT) of obese ORMDL3^TG^ male but not female mice (Supplemental Figs. 5A and D), whereas changes in macrophage infiltration were also observed in obese ORMDL3^TG^ female mice (Supplemental Figs. 5A and C). As visceral adiposity is associated with increased diabetes risk, while expansion of subcutaneous adipose tissue confers little or no risk [32], it is interesting to note that *Ormdl3* overexpression in obese mice leads to sexual dimorphism in visceral adipose remodelling, and less so in subcutaneous white adipose tissue.

### *3.4* *Ormdl3* overexpression in mice fed an obesogenic diet induces sexual dimorphism of visceral white adipose sphingolipids

**Figure 4:**
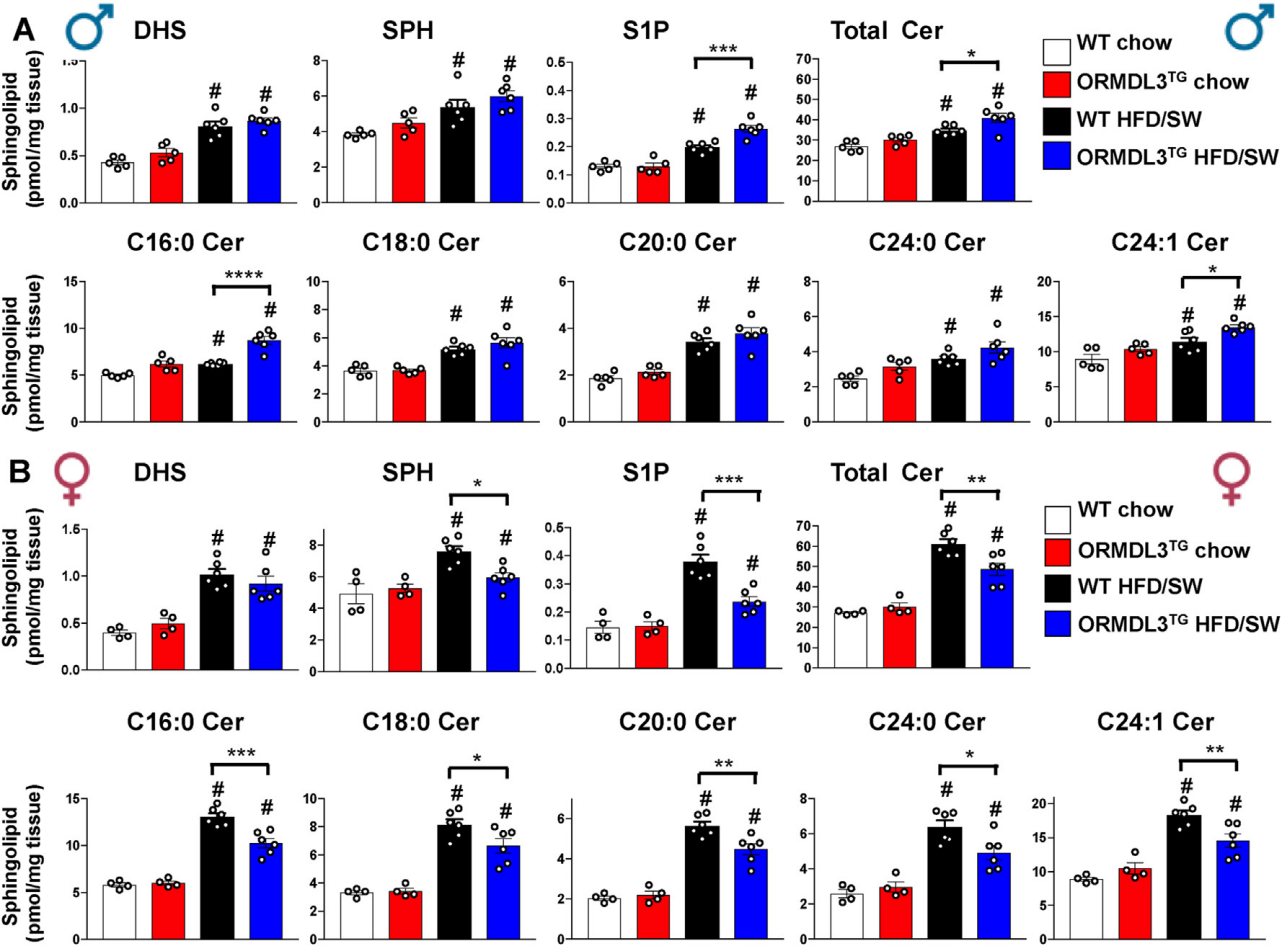
**Ceramides and S1P are increased in visceral adipose of obese males but decreased in female OMRDL3 overexpressing mice.** Sphingolipids were extracted from gWAT of WT and ORMDL3^TG^ male mice (A) and female mice (B) after 18 weeks of chow or HFD/SW feeding. Levels of sphingosine (SPH), dihydrosphingosine (DHS), sphingosine-1-phosphate (S1P), acyl chain ceramide (Cer) species and total Cer (including all ceramide species) were determined by liquid chromatography–electrospray ionization–tandem mass spectrometry. The numbers indicate the chain length followed by the number of double bonds in the fatty acid. Levels of the minor species C14:0Cer, C22:0Cer, C26:1Cer, and C26:0Cer are not shown. (N = 5,5,6,6 of male, N = 4,4,6,6 for female). Data are mean ± SEM. ^#^p ≤ 0.05 compared to corresponding littermate controls fed a chow diet. ∗p ≤ 0.05 compared to WT fed HFD/SW. Two-way analysis of variance test followed by Tukey's multiple comparison test.

In addition to other lipids, the key bioactive sphingolipid metabolites ceramide and sphingosine-1-phosphate (S1P) also accumulate in obesity and have been implicated in insulin resistance, diabetes, and NASH [8,9,[11], [12], [13],[33], [34], [35]]. Previous studies have shown that inhibition of SPT or deletion of *Sptlc2* alters the levels of ceramides accumulated in adipose depots in response to HFD feeding [8,12,36]. As ORMDL3 inhibits the catalytic activity of SPT, the rate-limiting step in *de novo* ceramide biosynthesis, we next assessed the effects of its overexpression on the sphingolipidome using liquid chromatography–electrospray ionization–tandem mass spectrometry. Consistent with previous studies [12,36,37], HFD/SW feeding increased the levels of all sphingolipid metabolites examined in the gWAT from both male and female WT mice (Figure 4A,B). As expected for a negative regulator of SPT, *Ormdl3* overexpression reduced the levels of total ceramides and S1P in HFD/SW-fed female mice (Figure 4B). Surprisingly, however, these sphingolipids were increased rather than decreased in the obese ORMDL3^TG^ male mice (Figure 4A). Notably, ORMDL3^TG^ male mice showed significantly elevated C16:0 ceramide, the predominant ceramide species in rodent adipose tissue, which correlated with weight gain and glucose intolerance [6,37]. On the other hand, ORMDL3^TG^ obese female mice had reduced levels of C16:0 ceramide, along with significant reductions in the levels of long- and very-long-chain ceramide species compared to obese female WT mice (Figure 4B). Thus, overexpression of ORMDL3 in obese mice appears to confer sexual dimorphism in the abundance of key sphingolipid metabolites S1P and ceramides in visceral adipose tissue.

### *3.5* *Ormdl3* overexpression induces hepatic steatosis, dyslipidemia, steatohepatitis, with progressive fibrosis in obese male but not female mice

**Figure 5:**
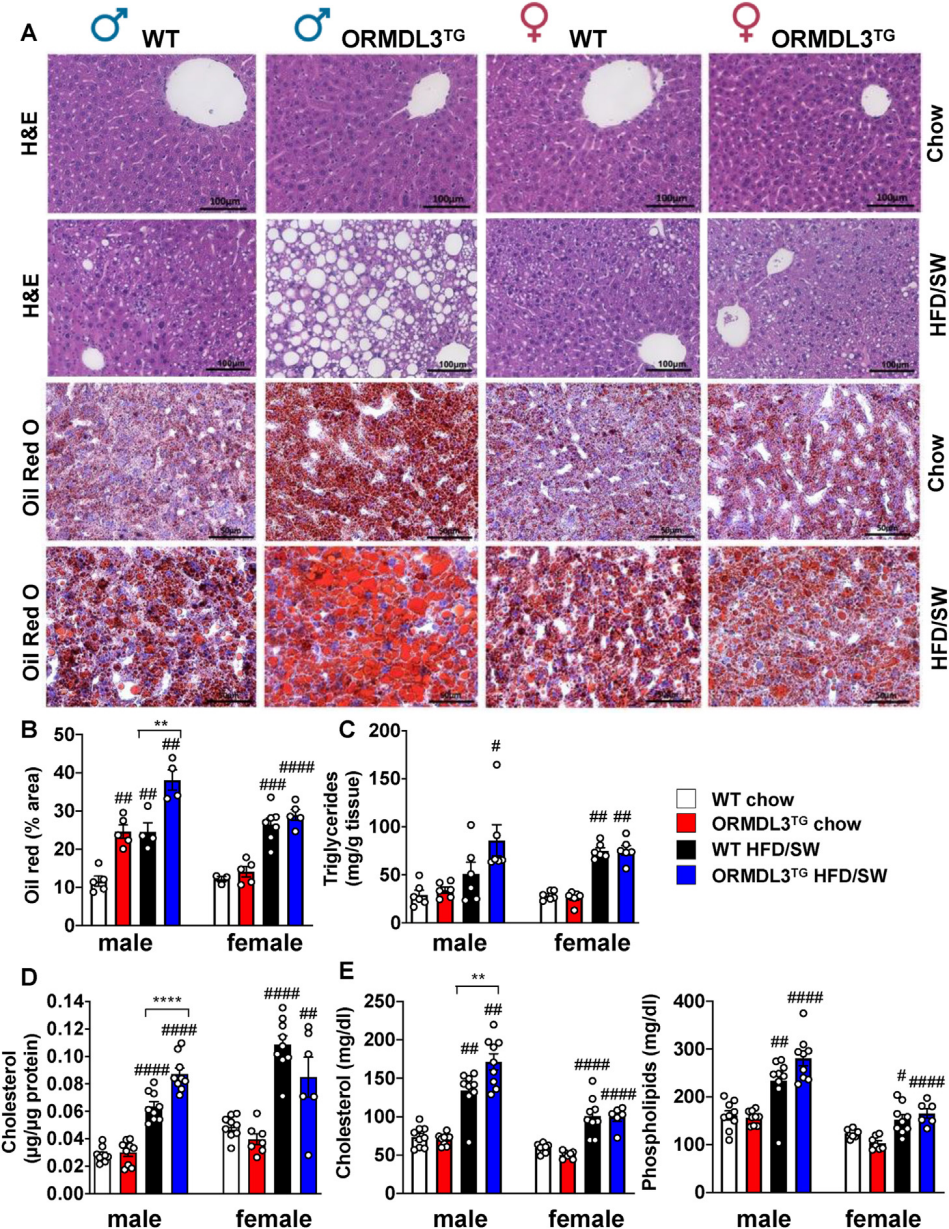
**Obese male but not female mice overexpressing ORMDL3 display increased hepatic steatosis and dyslipidemia.** (A) Representative images of H&E and Oil red O-stained liver sections from WT and ORMDL3^TG^ male and female mice fed chow or HFD/SW for 18 weeks as indicated (B) Oil red O staining was quantified as percent staining per total tissue surface area. (N = 3–7 mice per group) (C) Hepatic triglycerides (N = 6 mice per group). (D) hepatic cholesterol (N = 6–9 mice per group) E) plasma circulating cholesterol, and phospholipids (N = 6–9 mice per group). ^#^p ≤ 0.05 compared to corresponding littermate controls fed a chow diet. ∗p ≤ 0.05 compared to WT fed HFD/SW. Two-way analysis of variance test followed by Tukey's multiple comparison test.

Adipocyte expansion is finite, with excessive adipocyte hypertrophy leading to ectopic lipid accumulation in the liver and lipotoxicity [38]. Following the observation of aberrant adipocyte hypertrophy and macrophage infiltration in ORMDL3^TG^ male mice (Figure 3), we investigated their consequences on the liver. As expected, no macrovesicular lipid droplets were observed in either WT or ORMDL3^TG^ mice on chow diet, yet they were only clearly evident in ORMDL3^TG^ male mice but not in female mice fed HFD/SW (Figure 5A). Although ORMDL3 overexpression slightly increased Oil Red O staining of neutral lipids in male mice on chow diet, staining was much more pronounced when these mice were fed HFD/SW (Figure 5A,B). Increased steatosis was corroborated by increased hepatic triglycerides and cholesterol in obese ORMDL3^TG^ male mice, which were much greater than in obese WT males (Figure 5C,D). Importantly, HFD/SW fed ORMDL3^TG^ mice also had significantly elevated circulating levels of cholesterol and total phospholipids compared to obese male WT or obese ORMDL3^TG^ female mice (Figure 5E).

**Figure 6:**
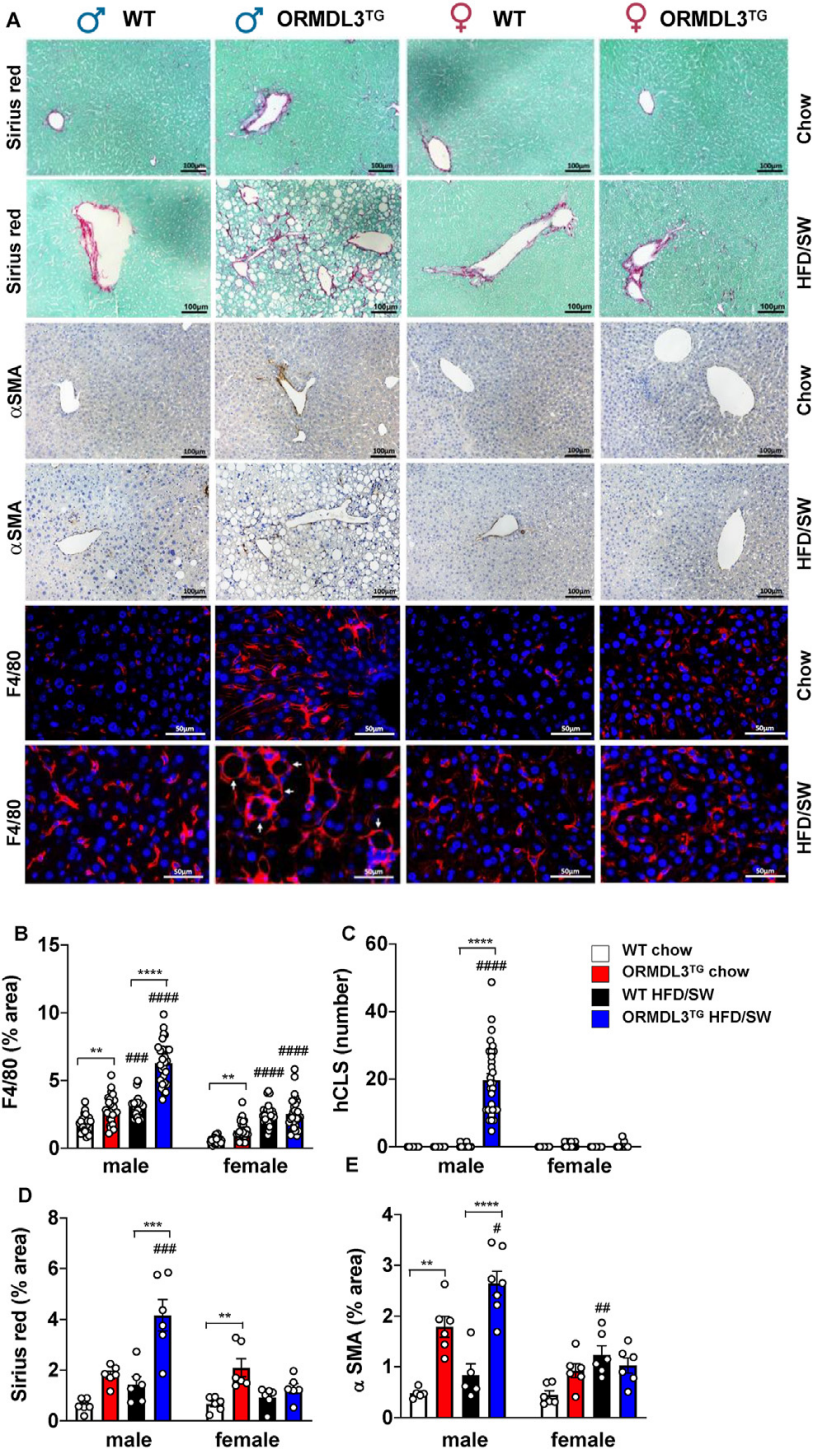
**Obese male but not female mice overexpressing ORMDL3 develop NASH.** (A) Representative images of liver sections from WT and ORMDL3^TG^ male and female mice fed chow or HFD/SW for 18 weeks stained with Sirius red/fast green, F4/80 or with anti-αSMA antibodies. (B) F4/80 staining quantified as percent staining per total tissue surface area and (C) numbers of hCLS per 1000 μm^2^ indicated by arrows in (A). (N = 5 mice per group with at least 25–30 different fields numerated). (D) Sirius red/fast green staining and αSMA staining were quantified as percent staining per total tissue surface area. (N = 5–7 mice per group). Data are mean ± SEM. ^#^p ≤ 0.05 compared to corresponding littermate controls fed a chow diet. ∗∗p ≤ 0.01 compared to WT fed HFD/SW. Two-way analysis of variance test followed by Tukey's multiple comparison test.

Immunohistochemical staining of the liver for F4/80, a marker for myeloid-derived macrophages that are recruited from the bone marrow and infiltrate the liver as part of an inflammatory process, and for tissue-resident Kupffer cells, indicated a robust increase in staining only in ORMDL3^TG^ male mice fed HFD/SW (Figure 6A,B). A scattered distribution of macrophages was observed in the livers of the obese WT mice (Figure 6A). In contrast, infiltrating macrophages in the livers of ORMDL3^TG^ obese male mice were clustered around large lipid droplets known as hepatic crown-like structures (hCLS) (Figure 6A,C). These unique histopathological features of human and murine NASH [39,40] were observed only in ORMDL3^TG^ male but not female mice (Figure 6C). It has been suggested that formation of hCLS precedes the development of collagen deposition and liver fibrosis [39] that are cardinal features of human NASH [41]. Indeed, Sirius red staining of liver sections revealed that only ORMDL3^TG^ male mice fed HFD/SW displayed significant amounts of collagen surrounding hepatic lipid droplets, whereas tissue fibrosis was rarely observed in the WT controls (Figure 6A,D). In contrast, *Ormdl3* overexpression in female mice fed with HFD/SW did not increase collagen staining (Figure 6A,D). Similarly, only obese male ORMDL3^TG^ mice and not obese female ORMDL3^TG^ mice showed significant liver deposition of αSMA, a marker of hepatic stellate cell activation (Figure 6E). Taken together, these results suggest that overexpression of ORMDL3 in obese mice bestows sexual dimorphism of hepatic steatosis, hypercholesterolemia, and exacerbated fibrosis, leading to the early development of NASH characteristics.

### 3.6 ORMDL3^TG^ male mice have elevated hepatic ER stress contributing to the development of NASH

**Figure 7:**
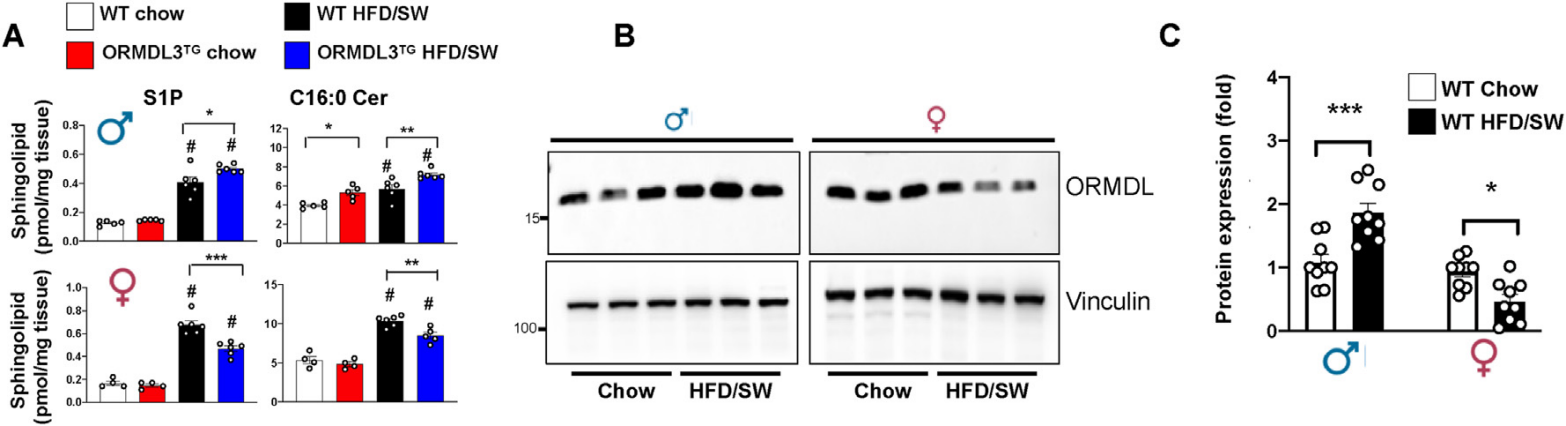
**Obesogenic diet induces ORMDL expression in male mice.** (A) Sphingolipids were extracted from livers of WT and ORMDL3^TG^ mice after 18 weeks of chow or HFD/SW feeding and levels S1P and C16:0 ceramide were determined by LC-ESI-MS/MS. Data are mean ± SEM (N = 4–6 mice per group). ^#^p ≤ 0.05 compared to corresponding littermate controls fed a chow diet. ∗p ≤ 0.05 compared to WT fed HFD/SW. Two-way analysis of variance test followed by Tukey's multiple comparison test. (B) Western blots of liver, from chow and HFD/SW fed male and female WT mice. Proteins were visualized with anti-ORMDL antibodies. Vinculin was used as loading controls. (C) Densitometric analyses of blots. (N = 9 mice per group). Mean ± SEM; ∗p ≤ 0.05 compared to corresponding littermate controls fed a chow diet. Two-way analysis of variance test followed by Tukey's multiple comparison test.

Next, it was of interest to examine the mechanism by which *Ormdl3* overexpression induced sex differences in the development of diet-induced non-alcoholic fatty liver disease and steatohepatitis in mice. Similar to white adipose tissue, sex differences in livers from ORMDL3^TG^ mice were observed in sphingolipid metabolites S1P and C16:0 ceramide, which are known to be involved in the pathogenesis of obesity, insulin resistance, and the development of NASH [6,8,9,12,13,33,35,37,42]. Whereas *Ormdl3* overexpression increased S1P and C16:0 ceramide in male mice, it reduced them in female mice (Figure 7A).

Although diet increased levels of other ceramide species, Ormdl3 overexpression did not affect their levels in males yet levels of C18:1 and C20:0 ceramides were reduced in females (Supplemental Fig. 6).

Consistent with our previous observation that larger increases in ORMDL3 expression, as seen in pathologies, antithetically increase ceramide formation rather than decreasing it [43,44], we observed that feeding a western diet significantly increased hepatic ORMDL protein levels in male mice and slightly decreased it in female mice (Figure 7B,C). Diet also increases the levels of the transgene ORMDL3-FLAG in males (Supplementary Fig. 2). Analysis of differentially expressed mRNAs among healthy controls and NASH patients using publicly available data (GSE89632) [45] revealed that hepatic expression of *ORMDL3*, but not *ORMDL1* or *ORMDL2*, were significantly upregulated in male but not female NASH samples compared to healthy controls. Moreover, genes encoding enzymes that regulate *de novo* ceramide biosynthesis including *SPTLC2*, were also significantly overexpressed in NASH male patients (Supplemental Fig. 7).

**Figure 8:**
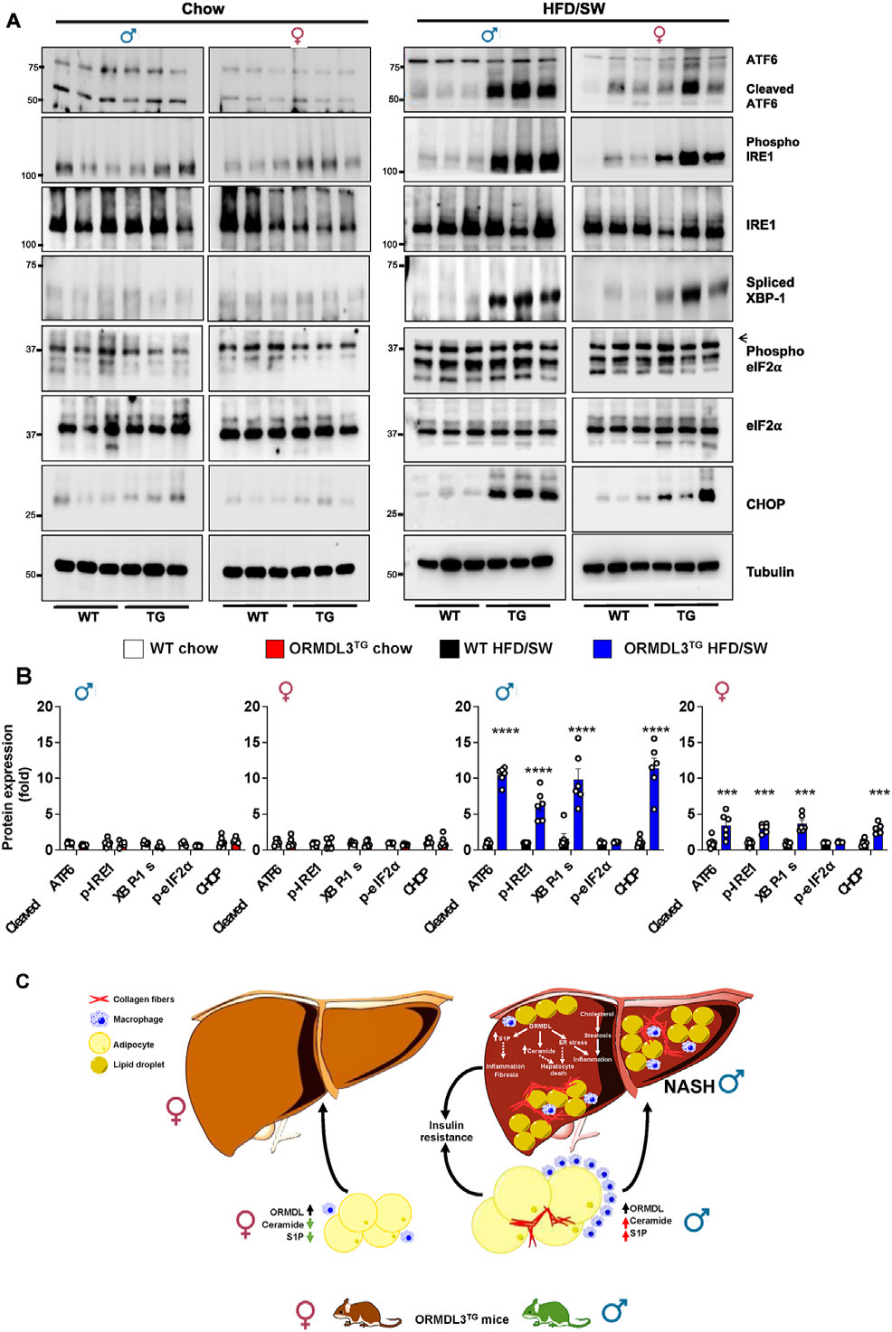
**ORMDL3 overexpression in male mice fed an obesogenic diet induces excessive hepatic ER stress and the unfolded protein response.** (A,B) Hepatic protein levels from WT and ORMDL3^TG^ mice fed Chow or HFD/SW for 18 weeks were analyzed for UPR by immunoblotting with the indicated antibodies. Tubulin was used as a loading control. Densitometric analysis of proteins involved in the UPR are shown in (B). (N = 6 mice per group). Data are mean ± SEM, ∗p ≤ 0.05 compared to WT fed HFD/SW. Two-way analysis of variance test followed by Tukey's multiple comparison test. Arrowhead indicates p-eIF2**. (C) Schematic depicting the effect of ORMDL3 overexpression on obese male and female mice.** In male mice fed HFD/SW diet, overexpression of ORMDL3 induces excessive ER stress, UPR-exacerbated obesogenic sphingolipid accumulation leading to NASH development. In female mice fed HFD/SW diet, overexpression of ORMDL3 induces marginal ER stress and reduces obesogenic sphingolipids. See text for more information.

Nevertheless, it was also suggested that the pathogenic role for ORMDL3 stems from its effect on ER stress and the unfolded protein response (UPR) [44,[46], [47], [48]]. Therefore, we sought to examine the effect of *Ormdl*3 expression on UPR sensors that can be directly activated by lipotoxic ER stress via three ER transmembrane signal transducers: activating transcription factor 6 (ATF6), protein kinase RNA-like ER kinase (PERK), and inositol-requiring enzyme 1 (IRE1) [49]. There was robust activation of ATF6 in livers from ORMDL3^TG^ males observed by the appearance of 50 kDa cleaved ATF6, a major UPR-specific transcription factor (10-fold increase of cleaved ATF6 in liver from ORMDL3^TG^ males vs. 3-fold in ORMDL3^TG^ female mice, all compared to their sex matched controls). Likewise, increased auto-phosphorylation at Ser^724^ of IRE1α that activates its RNase activity [50] by OMRDL3 overexpression was more pronounced in males (Figure 8A,B). Although there was no obvious PERK activation, as determined by the phosphorylation of eukaryotic translation initiation factor 2α (eIF2α) at ser^51^, *Ormdl3* expression in obese male mice induced an 11-fold increase in the UPR-regulated CCAAT-enhancer-binding protein homologous protein (CHOP), a pro-apoptotic transcription factor, whereas it was only increased 2-fold in the livers of obese female mice (Figure 8A,B). Taken together, the aberrant accumulation of the key obesogenic sphingolipids S1P and ceramides, together with excessive and sustained lipotoxic ER stress in the livers of ORMDL3^TG^ male but not female mice, correlated with the sexual dimorphism of diet-induced non-alcoholic steatohepatitis induced by *Ormdl3* overexpression.

## 4 Discussion

NASH is a sex-dimorphic disease with a higher prevalence in men than in women of childbearing age [5]. Sex-specific fat distribution in visceral deposits in men compared with fat storage in subcutaneous adipose tissue in women was suggested to contribute to the lower metabolic risk in women [5]. Similar sex differences were also found in rodent models of diet-induced obesity [51]; however, the mechanisms that underlie this difference are still not well understood.

In this study, we show that overexpression of *Ormdl3* in mice fed HFD/SW resulted in sexual dimorphism of the key sphingolipid metabolites ceramides and S1P in visceral adipose tissue and the liver, and in metabolic and pathological processes involved in the development of NASH. Although Ormdl3 overexpression did not induce major changes in male or female mice fed a chow diet, male but not female ORMDL3^TG^ mice on obesogenic diet had worsened white adipose hypertrophy accompanied by increased MCP-1, which contributes to macrophage infiltration into adipose tissue and fibrosis severity (Figure 8C). Adipose dysfunction, severe adipocyte hypertrophy, and chronic low-grade inflammation may contribute to severe hepatic steatosis, insulin resistance, and liver fibrosis observed only in obese male ORMDL3^TG^ mice (Figure 8C). The elevated hepatic cholesterol content in these mice correlated with the formation of hCLS, known to form around crystalized cholesterol in human and murine NASH livers, and distinguishes NASH from simple steatosis [39,40]. Together, these results suggest a potential role for ORMDL3 expression in inter-organ cross-talk that can maintains overall metabolic homeostasis in obesity and NASH development.

Our finding that high Ormdl3 overexpression in obese male but not female mice accelerates NASH development might be relevant to the sexual dimorphism in development of this disease in humans. Notable hepatic upregulation of *ORMDL3* was observed in men and not women NASH patients in comparison to healthy controls, whereas *ORMDL1* and *ORMDL2* did not show a significant change. Additionally, only in male NASH patients, there was a significant increase in expression of *SPTLC2*, a subunit of SPT, that regulates its activity and *de novo* ceramide biosynthesis and contributes to metabolic diseases [9,13,52]. Thus, elevated *ORMDL3* expression could pose a risk for the onset of NASH among obese males, potentially contributing to the higher prevalence of NASH in males compared to females.

Our study has some limitations. We started the high fat diet feeding immediately after weaning following protocols examining the role of ceramide in insulin resistance and hepatic steatosis [34] rather than after reaching maturity, about 8 weeks of age [20,53]. Thus, we cannot exclude possible interference of sexual maturation/endocrine changes during puberty, which may be affected by diet and influence our results.

In contrast to the expectation that high levels of ORMDL3 would reduce the levels of these sphingolipids in metabolically active tissues, consistent with its function as a negative regulator of SPT [15], surprisingly this was observed only in obese female and not in obese male ORMDL3^TG^ mice. This disparity could be due to the increase in hepatic ORMDL protein expression in males induced by the obesogenic diet which reduces ORMDL expression in females. This is consistent with previous observations that small increases in ORMDL expression, as expected, lead to a reduction in ceramide biosynthesis, whereas larger increases in ORMDL expression, as seen in many pathologies such as childhood asthma and atherosclerosis [54,55], cause increased rather than decreased ceramide levels, potentially intensifying disease progression [43,44]. The increase we observed in ceramides and especially C16:0 ceramide in obese male mice is particularly relevant, as expression of CerS6, which preferentially generates C16:0 ceramide, is increased in the adipose tissue and liver of obese mice and humans [37]. Moreover, liver CerS6 deletion or overexpression in mice suggests that C16:0 ceramide is a critical mediator of obesity and insulin resistance [37]. However, it should be noted that the C16:0 ceramides generated by CerS5 or CerS6 in different subcellular compartments have distinct functions. It was shown that in response to HFD, CerS6, but not CerS5, induces C16:0 ceramide accumulation in mitochondria [7]. Elevated C16:0 ceramide in turn specifically binds to mitochondrial fission factor, to regulate mitochondria fission and promote insulin resistance, obesity and steatosis in mice [7]. Differential regulation of hepatic ceramides in response to HFD/SW between the sexes of mice have been previously noted [56]. Similar to this report [56], we observed that WT female mice had higher levels of C16:0 ceramide and other ceramide species than males. Although the reason for this is not clear, it is tempting to speculate that this C16:0 ceramide in females is produced by CerS5 in distinct subcellular location that does not contribute to steatosis and insulin resistance [7], as it is known that location of ceramide accumulation in different organelles dictates its actions [9]. Alternatively, the fact that females are protected from NASH development despite having elevated ceramide levels could be explained by the dual opposing roles of ceramide proposed by Summers and colleagues [34]. It was suggested that in early stages of the disease when triglyceride stores are saturated and cellular energy needs are met, increasing ceramides promote lipid uptake and incorporation of fatty acids into triglycerides as well as mitochondria fatty acids oxidation, to protect cells from fatty acid lipotoxicity [52]. In later stages of NASH, increased *de novo* biosynthesis of ceramides stimulates hepatocyte apoptosis, ER stress, inflammatory cytokines production, and fibrosis [34]. Thus, we are hypothesizing that in females, increased ceramide levels from the diet are mainly protective, whereas increased *de novo* ceramide biosynthesis in males contributes to NASH development. Together, these studies support the notion that the physiological roles of certain hepatic ceramides differ between sexes and that the relationship between these sphingolipid species and pathology is more complex than currently envisioned and deserves further study.

Several previous studies have demonstrated a role of ORMDL3 in regulation of ER stress and UPR, particularly ATF6 [44,[46], [47], [48]]. UPR sensors can be directly activated by toxic lipids, known as lipotoxic stress, independent of the misfolding of proteins or the canonical proteotoxic stress [57]. In agreement, simultaneous accumulation of neutral lipids, cholesterol, ceramide, and S1P in the livers of ORMDL3^TG^ male but not female mice led to large inductions of the ATF6 and IRE1α arms of the UPR and subsequent CHOP activation. This is consistent with another report that ATF6 can be activated directly by binding sphingolipids to a distinct motif in its transmembrane domain, which preferentially upregulates ER lipid biosynthetic genes [58]. Conversely, the UPR can also alter sphingolipid metabolism. For example, palmitate activates IRE1 in hepatocytes, which in turn upregulates SPTLC1 and increases ceramide levels [59]. Thus, a positive feedback loop may be created by hepatic sphingolipid accumulation to increase lipotoxicity-mediated sustained ER stress or maladaptive UPR that correlates with the sexual dimorphism of diet-induced development of steatosis and progression to NASH enhanced by *Ormdl3* overexpression. The roles of ORMDL3 in UPR and ER stress and in regulation of ceramide homeostasis are not mutually exclusive suggesting that both can contribute to the development of NASH in obese ORMDL3^TG^ male but not female mice.

In summary, this study uncovered an important role for ORMDL3 in sexual dimorphism in mice, which is important for the development and progression of NASH. Our findings suggest that high levels of ORMDL3 may be a risk factor for the development of NASH in obese males and is potentially relevant to how NASH disproportionately affects more males than females.

## Contributors

R.D.R.B and S.S designed the study and wrote the manuscript; R.D.R.B analysed data; R.D.R.B, C.D.G and C.W performed experiments; B.N and F.S.C provided expertise for metabolic phenotyping; R.L.P provided the mouse model. All authors reviewed and approved the final manuscript.

## Declaration of competing interest

The authors have declared that no conflict of interest exists.

## Data Availability

Data will be made available on request.
