# Supplementary material for: Overexpression of ORMDL3 confers sexual dimorphism in diet-induced non-alcoholic steatohepatitis
Source: Mol Metab. 2023 Dec 9;79:101851. doi: 10.1016/j.molmet.2023.101851 (PMC10772294; doi:10.1016/j.molmet.2023.101851)
Supplement: Multimedia component 1 [file mmc1.docx]

**Overexpression of ORMDL3 confers sexual dimorphism in diet-induced**

**non-alcoholic steatohepatitis**

**Ryan D.R. Brown^1^, Christopher D. Green^1^, Cynthia Weigel^1^, Bin Ni^2^,**

**Francesco S. Celi^2^, Richard L. Proia^3^, and Sarah Spiegel^1*^**

^1^Department of Biochemistry and Molecular Biology and ^2^Department of Internal Medicine, Virginia Commonwealth University School of Medicine, Richmond, VA, ^3^Genetics and Biochemistry Branch, National Institute of Diabetes and Digestive and Kidney Diseases, NIH, Bethesda, MD

**APPENDIX A: SUPPLEMENTAL MATERIAL**

**Supplemental Figure 1.** High expression of **Ormdl in metabolically active tissues of mice.** Western blots of Ormdl from liver, adipose tissue, lung, and kidney of mice. β-actin was used as a loading control**.**

**Supplemental Figure 2. Densitometric analyses of Western blots of ORMDL, FLAG, and UCP1.** Protein expression in liver (A), gWAT (B), sWAT (C), and BAT (D) from chow and HFD/SW fed WT and ORMDL3^TG^ male and female mice shown in Figure 1. Protein expression was normalized to Tubulin. For ORMDLs, total endogenous and ectopically expressed proteins were quantified. (N=6 per group). Data are mean ± SEM. *p≤0.05 compared to corresponding WT controls. Two-way analysis of variance test followed by Tukey's multiple comparison test.

**Supplemental Figure 3. Ormdl3 overexpression did not elicit changes in rectal body temperature.** Rectal temperature was determined in WT and ORMDL3^TG^ male and female mice (N=13,13, 15,16 for male, N=15,13,13,6 for female). Data are mean ± SEM. Three-way analysis of variance test followed by Tukey's multiple comparison test. No statistical differences between the groups.

**Supplemental Figure 4. ORMDL3 overexpression has minimal impact on food and water intake, respiration, and locomotor activity.** After 16 weeks of HFD/SW feeding, WT and ORMDL3^TG^ male and female mice were individually housed in metabolic chambers for 24 hours to acclimatize. Measurements for water consumption, food intake, energy balance, O_2_ consumption, CO_2_ production, and locomotor activity were measured during the 6 subsequent days and data displayed as average per day. (N=5-6 mice per group). Data are mean ± SEM.

^#^ p≤0.05 compared to ORMDL3^TG^ male. Two-way analysis of variance test followed by Tukey's multiple comparison test.

**Supplemental Figure 5. Increased adipocyte hypertrophy with worsened fibrosis in subcutaneous fat of obese male but not female ORMDL3^TG^ mice.** (A-E) WT and ORMDL3^TG^ male and female mice were fed chow or HFD/SW for 18 weeks. (N=5 mice per group). (A) Representative images of hematoxylin-eosin (H&E), Sirius red/fast green, and F4/80-stained subcutaneous adipose tissue (sWAT). (B) Adipocyte cell size. Data are mean ± SEM, ^#^ p≤0.05 compared to corresponding littermate controls fed a chow diet. *p≤0.05 compared to WT fed HFD/SW. One-way analysis of variance test followed by Tukey's multiple comparison test. (C) Fibrosis quantified as percent red staining per total tissue surface area. (N=5 mice per group). **p≤0.01 compared to WT fed HFD/SW, ^##^p≤0.01 compared to male ORMDL3^TG^. Two-way analysis of variance test followed by Tukey's multiple comparison test.

**Supplemental Figure 6. Effects of Ormdl3 expression on hepatic ceramide species of obese male and female mice.** Sphingolipids were extracted from liver of WT and ORMDL3^TG^ male mice (A) and female mice (B) after 18 weeks of chow or HFD/SW feeding. Levels of acyl chain ceramide (Cer) species were determined by liquid chromatography–electrospray ionization–tandem mass spectrometry. The numbers indicate the chain length followed by the number of double bonds in the fatty acid. Levels of the C16:0Cer are shown in Figure 7A and the minor species C14:0Cer, C22:0Cer, C26:1Cer, and C26:0Cer are not shown. Data are mean ± SEM (N= 4-6 mice per group). ^#^p≤0.05 compared to corresponding littermate controls fed a chow diet. *p≤0.05 compared to WT fed HFD/SW. Two-way analysis of variance test followed by Tukey's multiple comparison test.

**Supplemental Figure 7.** **Hepatic mRNA expression of ORMDL3 in human NASH.** Expression levels of ORMDL3 in 11 male and 13 female healthy controls and 9 male and 10 female NASH patients. Data was obtained from NCBI GEO (Dataset accession number GSE GSE89632) and are mean ± SEM. *p < 0.05. Two-way analysis of variance test followed by Tukey’s multiple comparisons test.

**Supplementary table 1**

| **Figure** | **Analysis** | **Sub analysis** | **Statistical test** | **Comparison and denotation** | **P value of factor or interaction** |
| --- | --- | --- | --- | --- | --- |
| 1b | RNA-seq of ORMDL across tissues | NA | Two-way ANOVA | Tissues compared to oesophagus (*) | Tissues compared to oesophagus - <0.0001 |
| 2a | Weight gain | Male | Three-way ANOVA | Factor 1 - Weeks on diet, Factor 2 - Diet (#), Factor 3 - Transgene (*) | F1 - <0.0001, F2 - <0.0002, F3 - NS, F1xF2 - 0.0001, F1xF3 - NS, F2xF3 - 0.0458, F1xF2xF3 - NS |
|  |  | Female | Three-way ANOVA | Factor 1 - Weeks on diet, Factor 2 - Diet (#), Factor 3 - Transgene (*) | F1 - <0.0001, F2 - <0.0001, F3 - NS, F1xF2 - 0.0001, F1xF3 - NS, F2xF3 - 0.0375, F1xF2xF3 - NS |
| 2b | Male organ weights | gWAT | Two-way ANOVA | Factor 1 - Transgene (*), Factor 2 - Diet (#) | F1 - NS, F2 - <0.0001, F1xF2 - NS |
|  |  | sWAT | Two-way ANOVA | Factor 1 - Transgene (*), Factor 2 - Diet (#) | F1 - NS, F2 - <0.0001, F1xF2 - NS |
|  |  | BAT | Two-way ANOVA | Factor 1 - Transgene (*), Factor 2 - Diet (#) | F1 - NS, F2 - <0.0001, F1xF2 - NS |
|  |  | Liver | Two-way ANOVA | Factor 1 - Transgene (*), Factor 2 - Diet (#) | F1 - NS, F2 - <0.0001, F1xF2 - NS |
| 2b | Female organ weights | gWAT | Two-way ANOVA | Factor 1 - Transgene (*), Factor 2 - Diet (#) | F1 - 0.0214, F2 - <0.0001, F1xF2 - 0.0025 |
|  |  | sWAT | Two-way ANOVA | Factor 1 - Transgene (*), Factor 2 - Diet (#) | F1 - 0.0363, F2 - <0.0001, F1xF2 - 0.0024 |
|  |  | BAT | Two-way ANOVA | Factor 1 - Transgene (*), Factor 2 - Diet (#) | F1 - NS, F2 - <0.0001, F1xF2 - 0.0280 |
|  |  | Liver | Two-way ANOVA | Factor 1 - Transgene (*), Factor 2 - Diet (#) | F1 - 0.0208, F2 - <0.0001, F1xF2 - NS |
| 2c | Male body composition | Fat mass | Two-way ANOVA | Factor 1 - Transgene (*), Factor 2 - Diet (#) | F1 - NS, F2 - <0.0001, F1xF2 - NS |
|  |  | Lean mass | Two-way ANOVA | Factor 1 - Transgene (*), Factor 2 - Diet (#) | F1 - NS, F2 - <0.0001, F1xF2 - NS |
| 2c | Female body composition | Fat mass | Two-way ANOVA | Factor 1 - Transgene (*), Factor 2 - Diet (#) | F1 - 0.0314, F2 - <0.0001, F1xF2 - 0.0038 |
|  |  | Lean mass | Two-way ANOVA | Factor 1 - Transgene (*), Factor 2 - Diet (#) | F1 - NS, F2 - 0.0223, F1xF2 - NS |
| 2d | Male GTT | NA | Two-way ANOVA | Factor 1 - Genotype (*), Factor 2 - Time | F1 - <0.0001, F2 - <0.0001, F1xF2 - <0.0001 |
| 2d | Female GTT | NA | Two-way ANOVA | Factor 1 - Genotype (*), Factor 2 - Time | F1 - NS, F2 - <0.0001, F1xF2 - NS |
| 2e | Male circulating insulin | NA | Two-way ANOVA | Factor 1 - Transgene (*), Factor 2 - Diet (#) | F1 - 0.0031, F2 - <0.0001, F1xF2 - 0.0310 |
| 2e | Female circulating insulin | NA | Two-way ANOVA | Factor 1 - Transgene (*), Factor 2 - Diet (#) | F1 - NS, F2 - 0.0186, F1xF2 - NS |
| 2f | Male HOMA-IR | NA | Two-way ANOVA | Factor 1 - Transgene (*), Factor 2 - Diet (#) | F1 - 0.0057, F2 - <0.0001, F1xF2 - NS |
| 2f | Female HOMA-IR | NA | Two-way ANOVA | Factor 1 - Transgene (*), Factor 2 - Diet (#) | F1 - NS, F2 - 0.0147, F1xF2 - 0.0246 |
| 3b | Male adipocyte size | NA | One-way ANOVA | comparing HFD/SW to chow (#), or ORMDL3TG to WT (*) | comparing HFD/SW to chow - <0.0001, or ORMDL3TG to WT - <0.0001 |
| 3b | Female adipocyte size | NA | One-way ANOVA | comparing HFD/SW to chow (#), or ORMDL3TG to WT (*) | comparing HFD/SW to chow - <0.0001, or ORMDL3TG to WT - <0.0001 |
| 3c | CSL | NA | Two-way ANOVA | Factor 1 - Transgene (*), Factor 2 - Gender (#) | F1 - <0.0001, F2 - 0.0007, F1xF2 - 0.0124 |
| 3d | MCP1 mRNA | NA | Two-way ANOVA | Factor 1 - Transgene (*), Factor 2 - Gender (#) | F1 - <0.0256, F2 - 0.0132, F1xF2 - 0.0132 |
|  | Acta mRNA | NA | Two-way ANOVA | Factor 1 - Transgene (*), Factor 2 - Gender (#) | F1 - <0.0315, F2 - 0.0289, F1xF2 - 0.0314 |
|  | Col1a1 mRNA | NA | Two-way ANOVA | Factor 1 - Transgene (*), Factor 2 - Gender (#) | F1 - NS, F2 - 0.0240, F1xF2 - 0.0242 |
| 3e | Sirius red staining | NA | Two-way ANOVA | Factor 1 - Transgene (*), Factor 2 - Gender (#) | F1 - 0.0212, F2 - 0.0107, F1xF2 - 0.0091 |
| 4a | gWAT male sphingolipids | DHS | Two-way ANOVA | Factor 1 - Transgene (*), Factor 2 - Diet (#) | F1 - NS, F2 - <0.0001, F1xF2 - NS |
|  |  | SPH | Two-way ANOVA | Factor 1 - Transgene (*), Factor 2 - Diet (#) | F1 - NS, F2 - 0.0001, F1xF2 - NS |
|  |  | S1P | Two-way ANOVA | Factor 1 - Transgene (*), Factor 2 - Diet (#) | F1 - 0.0041, F2 - <0.0001, F1xF2 - 0.0041 |
|  |  | C16:0 Cer | Two-way ANOVA | Factor 1 - Transgene (*), Factor 2 - Diet (#) | F1 - <0.0001, F2 - <0.0001, F1xF2 - 0.0253 |
|  |  | C18:0 Cer | Two-way ANOVA | Factor 1 - Transgene (*), Factor 2 - Diet (#) | F1 - NS, F2 - <0.0001, F1xF2 - NS |
|  |  | C20:0 Cer | Two-way ANOVA | Factor 1 - Transgene (*), Factor 2 - Diet (#) | F1 - NS, F2 - <0.0001, F1xF2 - NS |
|  |  | C24:0 Cer | Two-way ANOVA | Factor 1 - Transgene (*), Factor 2 - Diet (#) | F1 - 0.0096, F2 - 0.0002, F1xF2 - NS |
|  |  | C24:1 Cer | Two-way ANOVA | Factor 1 - Transgene (*), Factor 2 - Diet (#) | F1 - 0.0021, F2 - <0.0001, F1xF2 - NS |
|  |  | Total Cer | Two-way ANOVA | Factor 1 - Transgene (*), Factor 2 - Diet (#) | F1 - 0.0055, F2 - <0.0001, F1xF2 - NS |
| 4b | gWAT female sphingolipids | DHS | Two-way ANOVA | Factor 1 - Transgene (*), Factor 2 - Diet (#) | F1 - NS, F2 - <0.0001, F1xF2 - NS |
|  |  | SPH | Two-way ANOVA | Factor 1 - Transgene (*), Factor 2 - Diet (#) | F1 - NS, F2 - 0.0005, F1xF2 - 0.0206 |
|  |  | S1P | Two-way ANOVA | Factor 1 - Transgene (*), Factor 2 - Diet (#) | F1 - 0.0060, F2 - <0.0001, F1xF2 - 0.0037 |
|  |  | C16:0 Cer | Two-way ANOVA | Factor 1 - Transgene (*), Factor 2 - Diet (#) | F1 - 0.0067, F2 - <0.0001, F1xF2 - 0.0024 |
|  |  | C18:0 Cer | Two-way ANOVA | Factor 1 - Transgene (*), Factor 2 - Diet (#) | F1 - NS, F2 - <0.0001, F1xF2 - NS |
|  |  | C20:0 Cer | Two-way ANOVA | Factor 1 - Transgene (*), Factor 2 - Diet (#) | F1 - 0.0454, F2 - <0.0001, F1xF2 - 0.0126 |
|  |  | C24:0 Cer | Two-way ANOVA | Factor 1 - Transgene (*), Factor 2 - Diet (#) | F1 - NS, F2 - <0.0001, F1xF2 - 0.0232 |
|  |  | C24:1 Cer | Two-way ANOVA | Factor 1 - Transgene (*), Factor 2 - Diet (#) | F1 - NS, F2 - <0.0001, F1xF2 - 0.0044 |
|  |  | Total Cer | Two-way ANOVA | Factor 1 - Transgene (*), Factor 2 - Diet (#) | F1 - NS, F2 - <0.0001, F1xF2 - 0.0078 |
| 5b | Oil red O | Male | Two-way ANOVA | Factor 1 - Transgene (*), Factor 2 - Diet (#) | F1 - <0.0001, F2 - <0.0001, F1xF2 - NS |
|  |  | Female | Two-way ANOVA | Factor 1 - Transgene (*), Factor 2 - Diet (#) | F1 - NS, F2 - <0.0001, F1xF2 - NS |
| 5c | Liver TAG | Male | Two-way ANOVA | Factor 1 - Transgene (*), Factor 2 - Diet (#) | F1 - NS, F2 - 0.0025, F1xF2 - NS |
|  |  | Female | Two-way ANOVA | Factor 1 - Transgene (*), Factor 2 - Diet (#) | F1 - NS, F2 - <0.0001, F1xF2 - NS |
| 5d | Liver cholesterol | Male | Two-way ANOVA | Factor 1 - Transgene (*), Factor 2 - Diet (#) | F1 - 0.0005, F2 - <0.0001, F1xF2 - 0.0018 |
|  |  | Female | Two-way ANOVA | Factor 1 - Transgene (*), Factor 2 - Diet (#) | F1 - 0.0302, F2 - <0.0001, F1xF2 - NS |
| 5e | Circulating lipids | Male cholesterol | Two-way ANOVA | Factor 1 - Transgene (*), Factor 2 - Diet (#) | F1 - 0.0287, F2 - <0.0001, F1xF2 - 0.0098 |
|  |  | Female cholesterol | Two-way ANOVA | Factor 1 - Transgene (*), Factor 2 - Diet (#) | F1 - NS, F2 - <0.0001, F1xF2 - NS |
|  |  | Male phospolipids | Two-way ANOVA | Factor 1 - Transgene (*), Factor 2 - Diet (#) | F1 - NS, F2 - <0.0001, F1xF2 - NS |
|  |  | Female phospolipids | Two-way ANOVA | Factor 1 - Transgene (*), Factor 2 - Diet (#) | F1 - NS, F2 - <0.0001, F1xF2 - 0.0396 |
| 6b | F4/80 staining | Male | Two-way ANOVA | Factor 1 - Transgene (*), Factor 2 - Diet (#) | F1 - <0.0001, F2 - <0.0001, F1xF2 - <0.0001 |
|  |  | Female | Two-way ANOVA | Factor 1 - Transgene (*), Factor 2 - Diet (#) | F1 - 0.0059, F2 - <0.0001, F1xF2 - 0.0283 |
| 6c | hCLS | Male | Two-way ANOVA | Factor 1 - Transgene (*), Factor 2 - Diet (#) | F1 - <0.0001, F2 - <0.0001, F1xF2 - <0.0001 |
|  |  | Female | Two-way ANOVA | Factor 1 - Transgene (*), Factor 2 - Diet (#) | F1 - 0.0060, F2 - NS, F1xF2 - NS |
| 6d | Sirius red staining | Male | Two-way ANOVA | Factor 1 - Transgene (*), Factor 2 - Diet (#) | F1 - <0.0001, F2 - 0.0002, F1xF2 - 0.0460 |
|  |  | Female | Two-way ANOVA | Factor 1 - Transgene (*), Factor 2 - Diet (#) | F1 - 0.0008, F2 - NS, F1xF2 - 0.0027 |
| 6e | aSMA staining | Male | Two-way ANOVA | Factor 1 - Transgene (*), Factor 2 - Diet (#) | F1 - <0.0001, F2 - 0.0089, F1xF2 - NS |
|  |  | Female | Two-way ANOVA | Factor 1 - Transgene (*), Factor 2 - Diet (#) | F1 - NS, F2 - 0.0046, F1xF2 - 0.0231 |
| 7a | Liver sphingolipids | Male C16:0 Cer | Two-way ANOVA | Factor 1 - Transgene (*), Factor 2 - Diet (#) | F1 - 0.0002, F2 - <0.0001, F1xF2 - NS |
|  |  | Male S1P | Two-way ANOVA | Factor 1 - Transgene (*), Factor 2 - Diet (#) | F1 - 0.0166, F2 - <0.0001, F1xF2 - NS |
|  |  | Female C16:0 Cer | Two-way ANOVA | Factor 1 - Transgene (*), Factor 2 - Diet (#) | F1 - 0.0063, F2 - <0.0001, F1xF2 - NS |
|  |  | Female S1P | Two-way ANOVA | Factor 1 - Transgene (*), Factor 2 - Diet (#) | F1 - 0.0009, F2 - <0.0001, F1xF2 - 0.0040 |
| 7c | Densitometry | NA | Two-way ANOVA | Factor 1 - Diet (*), Factor 2 - Gender | F1 - NS, F2 - <0.0001, F1xF2 - <0.0001 |
| 7d | GSE database human gene expression | ORMDL1 | Two-way ANOVA | Factor 1 - NASH (*), Factor 2 - Gender | F1 - NS, F2 - NS, F1xF2 - NS |
|  |  | ORMDL2 | Two-way ANOVA | Factor 1 - NASH (*), Factor 2 - Gender | F1 - 0.0108, F2 - NS, F1xF2 - NS |
|  |  | ORMDL3 | Two-way ANOVA | Factor 1 - NASH (*), Factor 2 - Gender | F1 - NS, F2 - NS, F1xF2 - NS |
|  |  | SPTLC1 | Two-way ANOVA | Factor 1 - NASH (*), Factor 2 - Gender | F1 - NS, F2 - NS, F1xF2 - NS |
|  |  | SPTLC2 | Two-way ANOVA | Factor 1 - NASH (*), Factor 2 - Gender | F1 - 0.0030, F2 - NS, F1xF2 - NS |
|  |  | SPTssa | Two-way ANOVA | Factor 1 - NASH (*), Factor 2 - Gender | F1 - <0.0001, F2 - NS, F1xF2 - NS |
|  |  | SPTssb | Two-way ANOVA | Factor 1 - NASH (*), Factor 2 - Gender | F1 - NS, F2 - NS, F1xF2 - NS |
| 8b | Densitometry | Male chow | Two-way ANOVA | Factor 1 - Transgene (*), Factor 2 - Target protein | F1 - 0.0081, F2 - 0.0071, F1xF2 - NS |
|  |  | Male HFD/SW | Two-way ANOVA | Factor 1 - Transgene (*), Factor 2 - Target protein | F1 - <0.0001, F2 - <0.0001, F1xF2 - <0.0001 |
|  |  | Female chow | Two-way ANOVA | Factor 1 - Transgene (*), Factor 2 - Target protein | F1 - NS, F2 - NS, F1xF2 - NS |
|  |  | Female HFD/SW | Two-way ANOVA | Factor 1 - Transgene (*), Factor 2 - Target protein | F1 - <0.0001, F2 - 0.0014, F1xF2 - 0.0017 |
